# A Low-Complexity Near-Field Imaging Method for Multistatic Radar Systems Based on Receiver-Domain Decomposition

**DOI:** 10.3390/s26144471

**Published:** 2026-07-14

**Authors:** Anthony J. Weiss

**Affiliations:** School of Electrical Engineering and Computers, Tel Aviv University, Tel Aviv 6139001, Israel; tony@tauex.tau.ac.il

**Keywords:** near-field radar, multistatic radar, matched filtering, computational imaging, domain decomposition, interpolation, computational complexity, subaperture processing

## Abstract

Near-field multistatic radar imaging requires evaluating a nonlinear matched-filter operator over a three-dimensional search region, imposing a prohibitive computational burden on systems utilizing sparse, large-aperture receiver layouts. In this paper, we study a static-target formulation with a known signal envelope and develop a receiver-domain-decomposition for computation burden mitigation. Starting from a maximum-likelihood model, we show that when the temporal waveform is known, the estimation problem reduces to a coherent spatial matched filter formed from time-compressed data. This representation enables a direct comparison between brute-force image formation and an approximation in which the receiver set is partitioned into subapertures, low-resolution subimages are computed on a coarse spatial grid, corrected by a reference phase, interpolated to the fine grid, and coherently aggregated. We derive the matched-filter formulation, provide interpolation-based error bounds under compensated-image smoothness assumptions, and analyze computational complexity. Numerical simulations demonstrate that phase correction substantially smooths low-resolution block images, thereby enabling interpolation. The results also clarify the conditions under which the proposed approximation is accurate and where it is expected to degrade, including insufficient phase compensation, overly aggressive coarse-grid factors, and extended-target interference.

## Notation

Scalars are denoted by italic lowercase letters, vectors by bold lowercase letters, and matrices by bold uppercase letters. ${\left(\cdot \right)}^{T}$ and ${\left(\cdot \right)}^{H}$ denote transpose and conjugate transpose, respectively. ∥x∥ is the Euclidean ($L_{2}$) norm of vector x, and |·| denotes absolute value or complex modulus. Summary of principal notation is presented in Table 1.

## 1. Introduction

Near-field radar imaging differs fundamentally from its far-field counterpart because the propagation delay depends nonlinearly on the candidate spatial location. In multistatic systems with sparse or diluted receiver layouts, this nonlinearity is compounded by the fact that each receiver observes a distinct bistatic geometry. As a result, the corresponding matched-filter image must typically be evaluated directly over the search volume, which is computationally demanding even in static-target scenarios.

Backprojection or direct matched filtering remains the standard accuracy benchmark because it preserves the exact geometric phase model. However, its computational burden scales linearly with the number of receivers and image voxels and becomes prohibitive in large-scale problems. FFT-based accelerations are powerful when the forward model admits a global Fourier structure, but near-field bistatic propagation generally does not satisfy the assumptions needed for such a reduction [1,2]. Fast backprojection methods and related approximations can reduce cost, but they may introduce artifacts or inherit structural assumptions that are not well matched to sparse near-field multistatic layouts [3].

Boag proposed a multilevel domain-decomposition framework for radar imaging in which the data are partitioned into subdomains, low-resolution images are formed, phase corrected, and then interpolated and aggregated into a fine-resolution result [4]. That framework was developed in a different setting, with decomposition performed in a structured data domain. In the present work, the same principle is adapted to a revised imaging problem which leverages the inherent structure of the sparse receiver geometry rather than relying on traditional regular angle-frequency grids.

Compared with brute-force backprojection, the proposed method aims to approximate the same near-field matched-filter image while reducing the number of full-resolution voxel evaluations. Compared with per-receiver FFT fusion, the present method does not impose a global Fourier interpretation on the spatial geometry; rather, it preserves the exact spatial matched-filter structure and uses interpolation only after explicit phase correction. Compared with Boag’s original frequency-angle decomposition, the present formulation partitions the receiver domain into subapertures, which is the natural decomposition axis for sparse near-field multistatic arrays.

### 1.1. Contributions

The main contributions of this paper are as follows:

(1) A matched-filter derivation for a stationary target under known signal envelope, leading to a coherent spatial score formed from time-compressed data.
(2) A receiver-domain approximation in which coarse block images are phase corrected, interpolated, and coherently aggregated.
(3) A formal interpolation-error bound showing how approximation error depends on compensated-image smoothness and coarse-grid spacing.
(4) An analytical scalability extrapolation, based on the derived complexity model, showing that the achievable speedup is invariant to the imaging-volume size and improves monotonically with the number of receivers up to a bound set by the coarse-grid decimation factor.
(5) Numerical evidence demonstrating both the importance of phase correction and the regimes in which the approximation succeeds or fails.

### 1.2. Paper Organization

Section 2 reviews related work. Section 3 derives the radar model and matched-filter score for the stationary known-envelope case. Section 4 presents the proposed receiver-domain decomposition and phase-corrected interpolation method. Section 5 provides numerical results, including plots that explicitly show how phase correction smooths the low-resolution block images. Section 6 discusses computational complexity, an analytical scalability extrapolation, the interpolation error analysis, and the practical implications and limitations of the approach. Section 7 concludes the paper.

## 2. Related Work

Direct matched filtering and back projection form the conventional baseline for exact radar image formation because they preserve the underlying geometric model without resorting to asymptotic simplifications [1]. Their main drawback is computational cost. Fourier-based methods, including FFT-driven synthetic aperture radar reconstruction, are highly efficient when the imaging operator can be transformed into a Fourier form, but this generally relies on far-field approximations or other linearizing assumptions [2]. These approximations are often inaccurate in near-field multistatic problems. Matched filtering and operator-inversion formulations of near-field microwave imaging remain an active area, with recent work analyzing the conditions under which linearized inverse-scattering solutions are well posed for irregular near-field sampling [5]; the present paper adopts the matched-filter viewpoint but focuses on accelerating its evaluation rather than on the underlying inversion model.

Fast backprojection and related hierarchical accelerations have also been proposed [3]. A particularly influential example is the fast factorized back-projection (FFBP) algorithm, which recursively partitions the synthetic aperture and represents subimages in local polar coordinates, reducing complexity from $\Theta (N^{3})$ toward FFT-comparable cost while retaining a general aperture geometry [6]. More recent work has extended backprojection-type imaging to moving targets [7]. These methods reduce the number of operations while attempting to retain some of the physical fidelity of direct reconstruction. However, they may introduce local artifacts, smoothing, or geometry-dependent degradation, and, like FFBP, they are typically formulated for structured (e.g., linear or circular) aperture trajectories rather than for arbitrary sparse multistatic receiver layouts. The factorization idea has recently been extended specifically to near-field imaging with sparse MIMO arrays through the fast factorized Kirchhoff migration algorithm (FFKMA), which models the local spectral properties of near-field subimages to reduce sampling requirements while retaining applicability to generic, ultrasparse MIMO geometries [8]; a related sub-aperture polar-format algorithm has also been proposed for curved-trajectory millimeter-wave imaging [9]. Both are close contemporaries of the present work in that they also decompose the aperture into subsets and process each on a reduced grid before recombination. They differ from the present formulation primarily in their choice of intermediate representation: FFKMA operates in a locally defined spectral domain tailored to Kirchhoff migration, and the sub-aperture polar-format algorithm operates in polar image coordinates matched to a (near-)linear trajectory, whereas the present method keeps the block images in the original Cartesian imaging grid and relies solely on an analytic centroid-based reference phase, together with an explicit interpolation error bound, to make the coarse-to-fine step in each subaperture accurate.

Boag’s multilevel domain-decomposition method occupies a useful middle ground. Rather than forcing the data into a Fourier representation, it partitions the domain, computes low-resolution images, phase corrects them, and then interpolates and aggregates the results [4]. The same recursive decompose-interpolate-aggregate principle was originally developed for fast tomographic reprojection [10] and has since been applied across a range of high-frequency scattering and imaging problems. The present paper builds on that principle, but differs from Boag’s original formulation in several important respects.

Decomposition axis. Boag’s original method partitions the data domain along structured frequency-angle tiles, which is natural when the forward model is organized in the frequency-angle domain. In the present work, the decomposition is performed over disjoint receiver subsets (subapertures). This is the natural axis for sparse near-field multistatic arrays, where each receiver observes a geometrically distinct bistatic delay and there is no global frequency-angle structure to exploit.

Phase reference. In Boag’s framework, the reference phase is derived from the geometry of the frequency-angle tile. Here, the reference phase for each subaperture is defined using the centroid of the corresponding receiver subset, computed via the exact bistatic delay formula. This centroid-based reference accurately captures the dominant phase variation across the subaperture and is directly compatible with irregular or sparse receiver geometries. This use of a coarse reference phase to enable interpolation is conceptually related to, but distinct from, phase-correction techniques such as phase gradient autofocus, which estimate and remove an unknown phase error from the data itself rather than from known geometry [11]; in the present setting, the reference phase is computed analytically from the receiver-subset centroid, so no data-driven estimation step is required.

Signal model and time compression. The present formulation starts from a maximum-likelihood model with a known signal envelope, which allows the time dimension to be compressed into a single matched-filter vector before spatial processing begins. This time-compression step, which has no direct counterpart in Boag’s frequency-angle setting, simplifies the imaging operator and provides a clean baseline against which the approximation error can be formally quantified.

Error analysis. We derive explicit interpolation error bounds as a function of the coarse-grid spacing and the smoothness of the phase-compensated block images. This provides conditions under which the approximation is guaranteed to be accurate, and identifies the regimes—insufficient phase compensation, overly coarse grids, and extended-target interference—where it is expected to degrade.

Multistatic and distributed radar architectures, in which widely separated transmitters and receivers jointly observe a scene, provide spatial diversity that can improve detection, localization accuracy, and robustness to shadowing relative to a single monostatic aperture [12,13]. This diversity is a central motivation for the receiver-domain decomposition adopted here, since each subaperture in the present formulation corresponds to a distinct, geometrically compact view of the scene. The practical near-field consequences of such distributed sensing are well documented: multistatic near-field SAR with UAS-mounted apertures is known to suffer from strong, geometry-dependent sidelobe structure that must be actively suppressed [14]; multiview, multistatic, multifrequency ground-penetrating radar imaging increases both the achievable image quality and the data volume, or equivalently the reconstruction cost, that must be managed [15]; distributed MIMO radar operating under near-field wavefront curvature requires dedicated range–angle-coupled processing to avoid target energy loss [16]; and near-field terahertz imaging with non-uniform sparse multistatic arrays similarly requires dedicated fast-reconstruction strategies to remain tractable [17]. The present method is aimed at exactly this class of problems: sparse, irregularly distributed multistatic receivers observing a near-field scene, where the computational cost of coherent image formation grows with both the receiver count and the reconstruction volume.

Phase-aware interpolation, in which known or estimated propagation-phase terms are removed prior to interpolation and reinstated afterward, has also been used to control the accuracy of complex-valued image interpolation in conventional backprojection SAR [18]. This is conceptually close to the reference-phase compensation step of the present method, though it estimates and controls an already-known geometric phase term via a receiver-subset centroid, rather than an unknown phase error estimated from the data itself. Beyond conventional radar imaging, low-complexity near-field image-formation methods are increasingly relevant to integrated sensing and communication (ISAC) systems, which are expected to reuse distributed communication infrastructure and waveforms for sensing in beyond-5G and 6G networks and therefore inherit the same sparse-aperture, limited-compute constraints addressed here [19].

Near-field and multistatic imaging with sparse or distributed apertures has also received substantial attention outside the domain-decomposition literature. For sparse multiple-input multiple-output (MIMO) arrays, nonuniform-FFT range-migration algorithms exploit an approximate Fourier structure of the near-field forward model to avoid direct coherent summation [20], and multistatic MIMO configurations have also been used for high-resolution inverse-SAR imaging of large objects [21]. Compressive-sensing formulations instead exploit target sparsity to reconstruct near-field radar images from reduced measurements [22], and, more recently, deep-learning-based and plug-and-play reconstruction methods have been used to accelerate near-field MIMO imaging, either by learning a direct mapping from backprojected data to the reflectivity image [23] or by combining a physics-based data-fidelity term with a learned magnitude prior [24]. These approaches address a related but distinct problem: they primarily target densely sampled, structured (often planar) MIMO apertures and, in the compressive-sensing and learning-based cases, rely on statistical priors (sparsity or a training set) rather than on an explicit, geometry-based approximation error bound. In contrast, the present work targets sparse, irregularly distributed multistatic receiver layouts and provides a deterministic, analytically justified error bound that does not require sparsity assumptions on the scene or a training stage.

## 3. Radar Model and Matched-Filter Derivation

**Figure 1:**
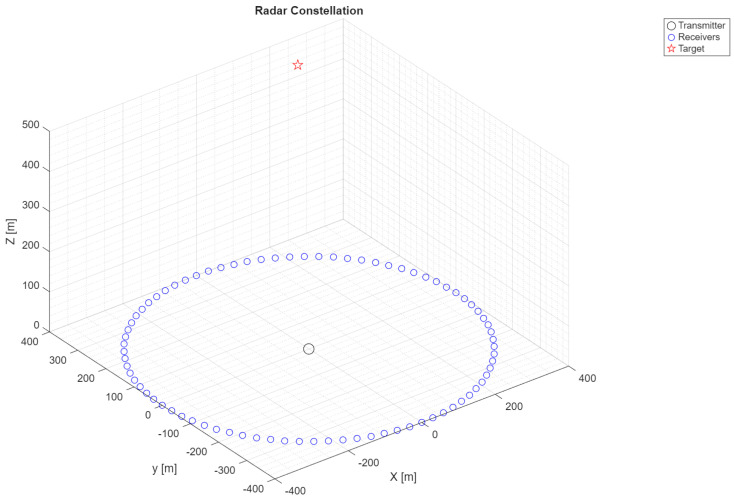
Potential system layout (used in the simulations).

Consider a transmitter located at $p_{tx}\in R^{3}$ and a set of *M* receivers at locations $q_{m}\in R^{3}$, m=1,…,M. See Figure 1. We assume a stationary point target at position $p\in R^{3}$. The bistatic delay for the *m*-th receiver is

(1) $$\tau_{m}\left(p\right)=\frac{∥p-p_{tx}∥+∥p-q_{m}∥}{c},$$

where *c* is the propagation speed and ∥x∥ is the $L_{2}$ norm of the vector x.

Let d[n] denote the known signal envelope, $f_{c}$ the carrier frequency, and α an unknown complex amplitude. Under the narrowband assumption, the received data at receiver *m* and sample index *n* are modeled as

(2) $$x_{m}\left[n\right]=\alpha \;d\left[n\right]\;e^{-j2\pi f_{c}\tau_{m}\left(p\right)}+w_{m}\left[n\right],$$

where $w_{m}\left[n\right]$ denotes additive complex Gaussian noise. Define the vectors

(3) $$x\left[n\right]={\left[\begin{array}{ccc} x_{1}\left[n\right] & \ldots & x_{M}\left[n\right] \end{array}\right]}^{T}.$$

(4) $$a\left(p\right)={\left[\begin{array}{ccc} e^{-j2\pi f_{c}\tau_{1}\left(p\right)} & \ldots & e^{-j2\pi f_{c}\tau_{M}\left(p\right)} \end{array}\right]}^{T}.$$

(5) $$w\left[n\right]={\left[\begin{array}{ccc} w_{1}\left[n\right] & \ldots & w_{M}\left[n\right] \end{array}\right]}^{T}.$$

Here a(p) is the *steering vector*, whose *m*-th element $e^{-j2\pi f_{c}\tau_{m}\left(p\right)}$ encodes the bistatic phase delay to the candidate point p at receiver *m*. Using these vectors, the observed signal equation becomes,

(6) x[n]=αa(p)d[n]+w[n],

A matched filter for this signal is

(7) $$Q\left(\hat{p}\right)={\left|a^{H}\left(\hat{p}\right)\sum_{n=1}^{N}\bar{d}\left[n\right]x\left[n\right]\right|}^{2}.$$

Let the measurements be organized into a matrix $X\in C^{M\times N}$ whose (m,n)-th element is $x_{m}\left[n\right]$. Because the signal envelope is known, the time dimension can be compressed into a single vector

(8) $$y=X\bar{d},$$

where $d={\left[d\left[0\right],d\left[1\right],\ldots ,d\left[N-1\right]\right]}^{T}$ and the overline denotes complex conjugation.

The matched-filter score is then

(9) $$Q\left(\hat{p}\right)={\left|a^{H}\left(\hat{p}\right)y\right|}^{2}.$$

The direct image is formed by evaluating (9) over all candidate voxels. As shown in the sequel in our setting, the size of a typical voxel is about 10 cm in each of the three dimensions (*x*, *y*, *z*). Thus, to cover a volume of a cubic km, we need $10^{12}$ voxels. This motivates our search for computation load reduction.

## 4. Receiver-Domain Approximation

**Figure 2:**
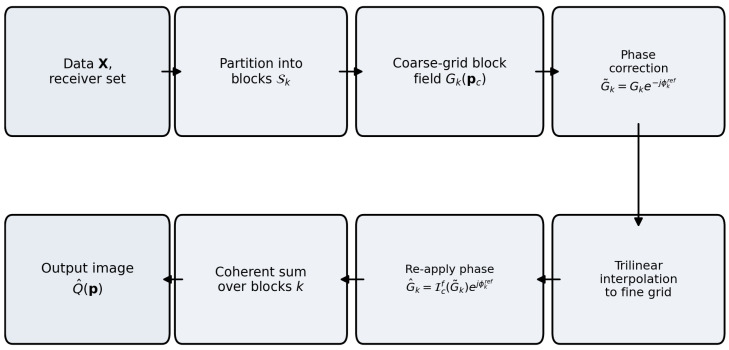
Block diagram of the proposed receiver-domain approximation. Data from each receiver subaperture are imaged on a coarse grid, phase corrected using the subaperture centroid, interpolated to the fine grid, rephased, and coherently combined across blocks.

This section presents the proposed method in full. The receiver partition, the coarse-grid block imaging step, the reference-phase correction, and the interpolation-and-aggregation procedure detailed in the four subsections below together constitute the receiver-domain approximation introduced in this paper; no part of this section is drawn from prior work. Figure 2 summarizes the resulting processing pipeline, from the raw receiver data to the reconstructed fine-grid image.

### 4.1. Receiver Partition

Let the receiver set be partitioned into *K* disjoint subsets,

(10) $$\left\{1,\ldots ,M\right\}=\overset{K}{\underset{k=1}{⋃}}S_{k}.$$

Correspondingly, define block steering vectors $a_{k}\left(p\right)$ and block-compressed data $y_{k}$. Then the complex matched field is

(11) $$G\left(p\right)=a^{H}\left(p\right)y=\sum_{k=1}^{K}G_{k}\left(p\right),\;G_{k}\left(p\right)=a_{k}^{H}\left(p\right)y_{k}.$$

The score is $Q\left(p\right)={\left|G\left(p\right)\right|}^{2}$.

### 4.2. Coarse-Grid Subimages

Instead of evaluating each block field on the fine grid $\Omega_{f}$, we compute it on a coarse grid $\Omega_{c}$: a suitable fine grid may use 8 points per voxel, while a suitable coarse grid may use 1 point per voxel.

(12) $$G_{k}\left(p_{c}\right),\;p_{c}\in \Omega_{c}.$$

### 4.3. Reference Phase Correction

Let ${\bar{q}}_{k}$ denote the centroid of the receiver subset $S_{k}$. Define the reference delay

(13) $$\tau_{k}^{\mathrm{ref}}\left(p\right)=\frac{∥p-p_{tx}∥+∥p-{\bar{q}}_{k}∥}{c},$$

and the corresponding reference phase

(14) $$\phi_{k}^{\mathrm{ref}}\left(p\right)=2\pi f_{c}\tau_{k}^{\mathrm{ref}}\left(p\right).$$

The compensated coarse block image is

(15) $${\tilde{G}}_{k}\left(p_{c}\right)=G_{k}\left(p_{c}\right)e^{-j\phi_{k}^{\mathrm{ref}}\left(p_{c}\right)}.$$

This removes the dominant oscillatory component and is the key step that enables stable interpolation.

### 4.4. Interpolation and Rephasing

Let $I_{c}^{f}$ denote trilinear interpolation from the coarse to the fine grid. The reconstructed block field on the fine grid is

(16) $${\hat{G}}_{k}\left(p\right)=I_{c}^{f}\;\left({\tilde{G}}_{k}\right)\left(p\right)e^{j\phi_{k}^{\mathrm{ref}}\left(p\right)}.$$

The full approximation is then

(17) $$\hat{Q}\left(p\right)={\left|\sum_{k=1}^{K}{\hat{G}}_{k}\left(p\right)\right|}^{2}.$$

## 5. Numerical Results

### 5.1. Simulation Setup

The numerical experiments use a single transmitter at the origin and M=80 receivers uniformly distributed on a circular ring of radius 400 m in the plane z=0. The target is stationary and located at

(18) $$p_{★}={\left[200,\;300,\;500\right]}^{T}\;m.$$

The carrier frequency is 2 GHz, and the known signal envelope is used for time compression. The receiver set is partitioned into K=4 equal-sized angular blocks around the ring, and the coarse grid uses decimation factors $\gamma_{x}=\gamma_{y}=\gamma_{z}=2$. This choice is not arbitrary: Section 6 (Discussion) shows that the interpolation error bound scales quadratically with the coarse-grid spacing and grows with the residual phase deviation across each subaperture, so *K* and γ trade off directly against the achievable speedup. With M=80 receivers, K=4 blocks keep each subaperture angularly compact (20 receivers, a $90^{\circ }$ arc) so that the centroid-based reference phase remains an accurate model of the block’s delay variation, while γ=2 keeps the coarse grid fine enough that the $C^{2}$-smoothness assumption underlying the error bound holds without visible degradation, as confirmed by the phase-gradient-energy reduction reported below. Coarser choices of either parameter increase speedup at the cost of accuracy, as quantified in Section 6.

### 5.2. Direct Versus Approximation Runtime

For a grid of 201×201×21 = 848,421 voxels, the measured runtime of the direct matched filter was

(19) $$T_{\mathrm{dir}}=9.142\;s,$$

while the approximation required

(20) $$T_{\mathrm{app}}=3.986\;s.$$

In this toy example, the speedup is a factor of 2.293. Both methods recover the correct target position.

### 5.3. Phase-Correction Smoothing Effect

**Figure 3:**
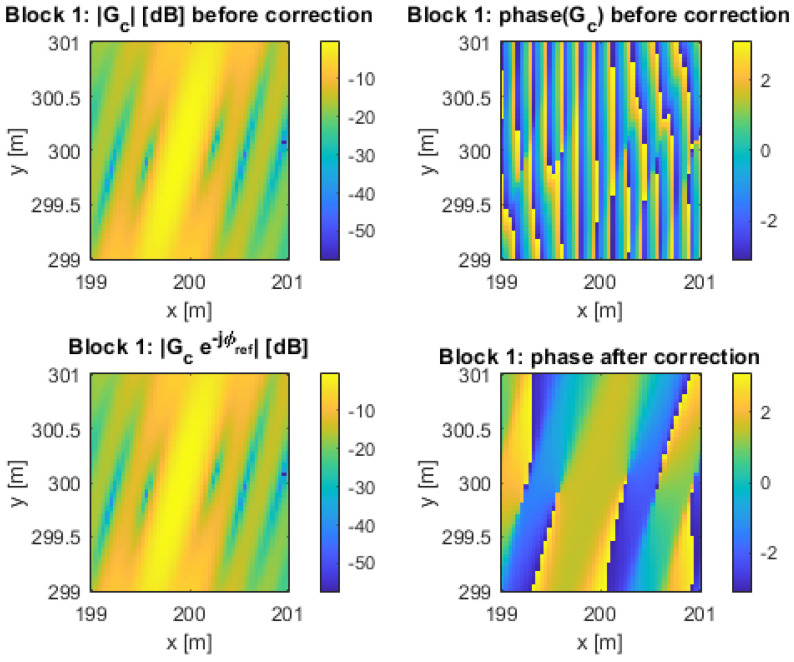
Representative coarse block image before and after phase correction. The corrected phase map is visibly smoother.

**Figure 4:**
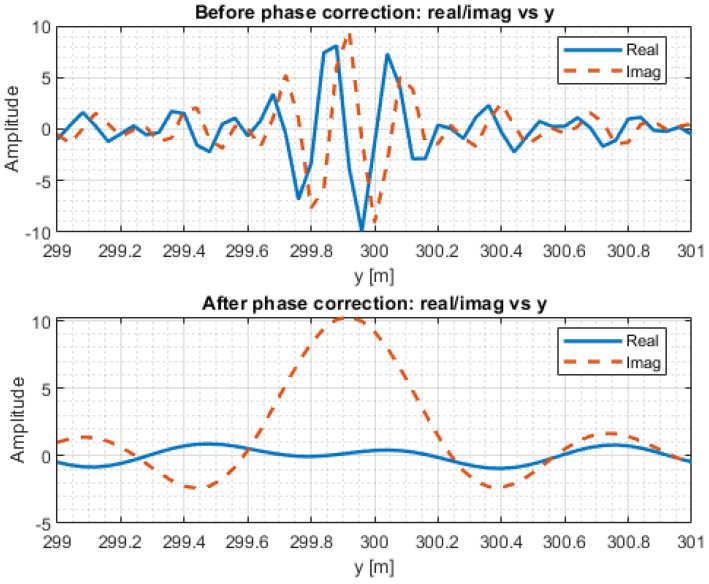
One-dimensional cuts of the coarse block field before and after phase correction, illustrating reduced oscillation after compensation.

The most important qualitative diagnostic is the low-resolution block image before and after phase correction. Figure 3 and Figure 4 show for a representative receiver block:

1. Magnitude and phase of the coarse block field before correction;
2. Magnitude and phase after multiplying by $e^{-j\phi_{k}^{\mathrm{ref}}}$;
3. One-dimensional cuts of the real and imaginary parts before and after correction.

These plots show that the uncompensated block image is highly oscillatory, while the corrected image varies much more slowly. This qualitative observation is confirmed by the phase-gradient-energy metric computed on the coarse block image. In the reported experiment, the phase-gradient energy decreased from

(21) $$E_{\phi ,\mathrm{before}}=3.15104$$

before correction to

(22) $$E_{\phi ,\mathrm{after}}=0.870591$$

after correction, corresponding to a reduction factor of

(23) $$\frac{E_{\phi ,\mathrm{before}}}{E_{\phi ,\mathrm{after}}}=3.619.$$

This confirms quantitatively that the reference-phase compensation substantially smooths the low-resolution block image and improves its suitability for interpolation. This is the central mechanism that makes interpolation plausible.

### 5.4. Score Cuts Along the Coordinate Axes

**Figure 5:**
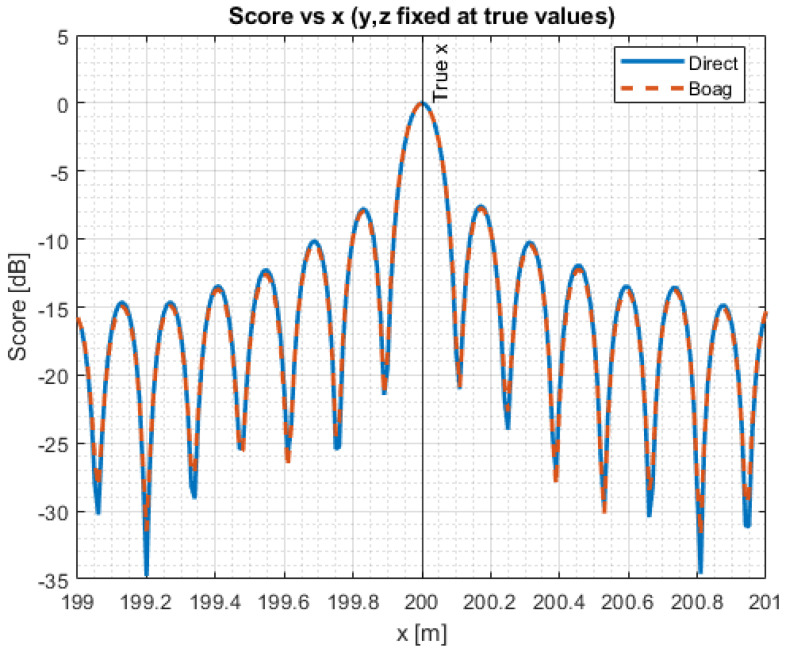
Normalized score in dB versus *x*, with *y* and *z* fixed at their true values.

**Figure 6:**
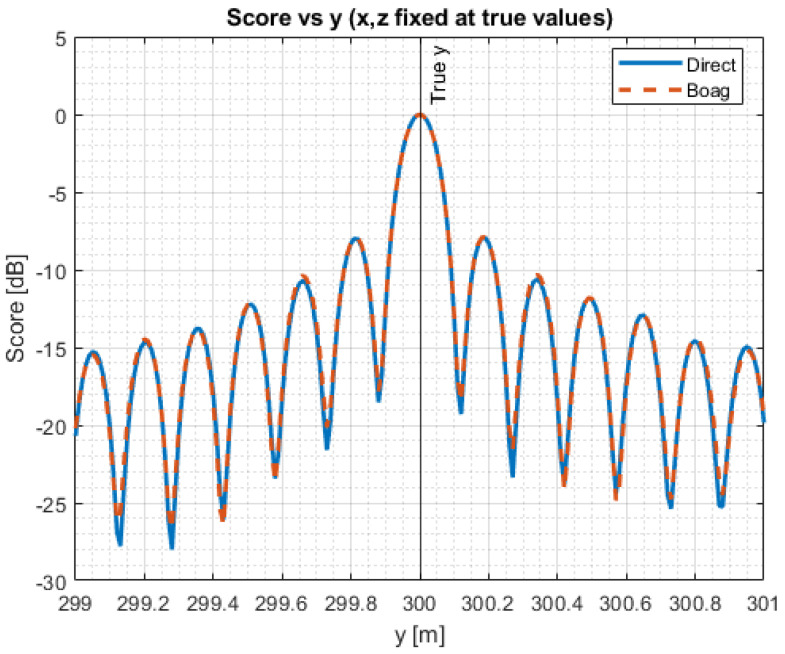
Normalized score in dB versus *y*, with *x* and *z* fixed at their true values.

**Figure 7:**
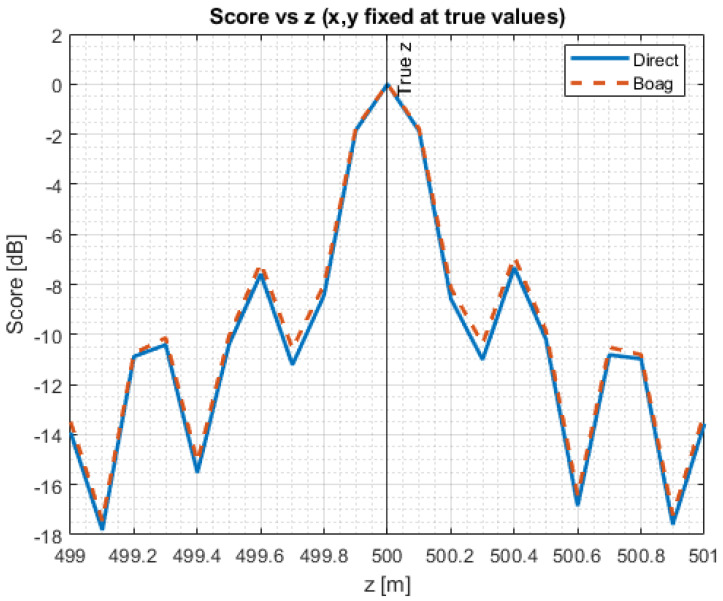
Normalized score in dB versus *z*, with *x* and *y* fixed at their true values.

To evaluate the impact of the approximation on localization structure, one-dimensional score cuts are plotted along the *x*, *y*, and *z* axes while fixing the remaining coordinates at the true target location. The scores are normalized and shown in dB. Figure 5, Figure 6 and Figure 7 reveal whether the approximation preserves the peak location and the local curvature of the matched-filter surface.

### 5.5. Accuracy Measures

The approximation can be quantified using normalized RMS and maximum error relative to the direct image

(24) $$\Delta_{\mathrm{rms}}=\frac{∥\hat{Q}{-Q∥}_{2}}{{∥Q∥}_{2}},\;\Delta_{\max}=\frac{∥\hat{Q}{-Q∥}_{\infty }}{{∥Q∥}_{\infty }}.$$

In the reported experiment with K=4 receiver blocks and decimation factor $\gamma_{x}=\gamma_{y}=\gamma_{z}=2$, the measured errors were $\Delta_{\mathrm{rms}}=0.089$ and $\Delta_{\max}=0.31$. The peak location was preserved exactly even when global image differences remained non-negligible, indicating that the approximation can remain useful for localization despite score-surface distortion away from the maximum.

### 5.6. Complexity–Accuracy Tradeoff

**Figure 8:**
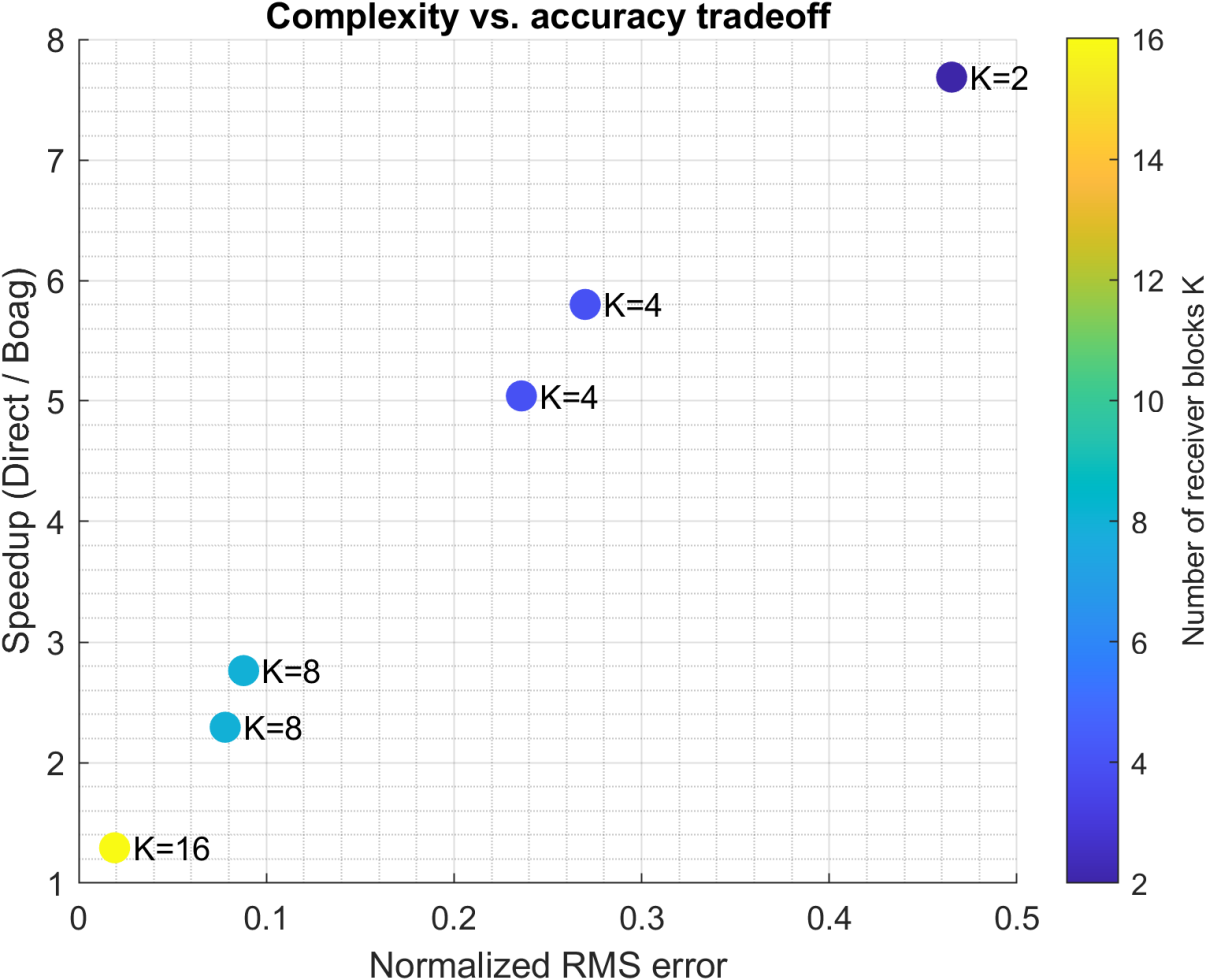
Complexity–accuracy tradeoff for the measured operating point, shown as speedup versus normalized RMS error. Additional sweep results can be added to reveal the full tradeoff frontier.

To summarize the tradeoff between computational efficiency and approximation fidelity, Figure 8 presents the measured speedup as a function of the normalized RMS error for the considered transmitter–receiver–target geometry. As expected, higher computational savings are accompanied by increased approximation error, illustrating the fundamental tradeoff between reduced evaluation time and interpolation accuracy.

## 6. Discussion

This section discusses the computational complexity of the proposed method, quantifies and interprets its interpolation error, and synthesizes the practical implications of the analysis and numerical results presented in Section 4 and Section 5.

### 6.1. Computational Complexity

Let $P=N_{x}N_{y}N_{z}$ denote the number of fine-grid voxels. Direct evaluation of (9) requires

(25) $$T_{\mathrm{dir}}=\Theta \left(MP\right).$$

If the coarse-grid decimation factors are $\left(\gamma_{x},\gamma_{y},\gamma_{z}\right)$, the coarse grid contains approximately

(26) $$P_{c}\approx \frac{P}{\gamma_{x}\gamma_{y}\gamma_{z}}.$$

The approximation requires evaluating all receiver blocks on the coarse grid plus interpolation on the fine grid

(27) $$T_{\mathrm{app}}=\Theta \;\left(MP_{c}+P\right).$$

Thus the potential gain appears only when the reduction in coarse-grid evaluations outweighs the interpolation overhead.

### 6.2. Analytical Scalability Extrapolation

The numerical experiments in Section 5 use a single, fixed-size configuration (M=80 receivers, P=848,421 fine-grid voxels), which already represents a substantial 3-D near-field imaging problem. Rather than re-running the simulation at additional sizes, this subsection uses the complexity model derived above, together with the single measured operating point, to characterize how the speedup is expected to behave as the problem grows. This is a model-based extrapolation, not new measured data, and its quantitative accuracy is bounded by the validity of the Θ(·) model and the calibration assumption stated below; we present it to characterize the qualitative trend rather than to assert exact numbers for untested configurations.

Write $T_{\mathrm{dir}}=c_{\mathrm{dir}}MP$ and $T_{\mathrm{app}}=c_{\mathrm{blk}}MP_{c}+c_{\mathrm{interp}}P$, where $c_{\mathrm{dir}}$, $c_{\mathrm{blk}}$, and $c_{\mathrm{interp}}$ are implementation-dependent per-operation constants and $P_{c}=P/\Gamma$ with $\Gamma =\gamma_{x}\gamma_{y}\gamma_{z}$ the total volumetric decimation factor (Γ=8 in the reported experiment). The predicted speedup is then

(28) $$S\left(M,P\right)\;=\;\frac{T_{\mathrm{dir}}}{T_{\mathrm{app}}}\;=\;\frac{c_{\mathrm{dir}}M}{\frac{c_{\mathrm{blk}}}{\Gamma }M+c_{\mathrm{interp}}}.$$

Two structural properties follow directly from (28), independent of the unknown constants:

(i) Invariance to grid size. *P* cancels out of the ratio entirely: for fixed *M* and Γ, the predicted speedup does not depend on the number of fine-grid voxels. This is because both $T_{\mathrm{dir}}$ and $T_{\mathrm{app}}$ scale linearly in *P* under the stated complexity model. In other words, the model predicts that the 2.29× speedup measured at P=848,421 voxels should persist, to leading order, at substantially larger imaging volumes; growing the search volume alone is not expected to erode the relative gain.

(ii) Monotonic, bounded scaling in *M*. As a function of *M* (with *P* and Γ fixed), S(M,P) is monotonically increasing, from S→0 as M→0 (the fixed interpolation overhead dominates when there are very few receivers) to a hard ceiling

(29) $$S\left(M,P\right)\;\overset{M\to \infty }{\to }\;\frac{c_{\mathrm{dir}}}{c_{\mathrm{blk}}}\;\Gamma \;\leq \;\Gamma ,$$

where the last inequality holds under the natural assumption $c_{\mathrm{dir}}≲c_{\mathrm{blk}}$ (the coarse-grid block evaluation performs the same type of coherent summation as the direct matched filter, so its per-voxel cost is comparable to or somewhat higher than the direct computation’s). That is, the achievable speedup cannot exceed the volumetric decimation factor Γ, and adding receivers can only move the operating point toward this ceiling, never away from it: more receivers are predicted to be at least as favorable for the proposed method, never less favorable.

**Figure 9:**
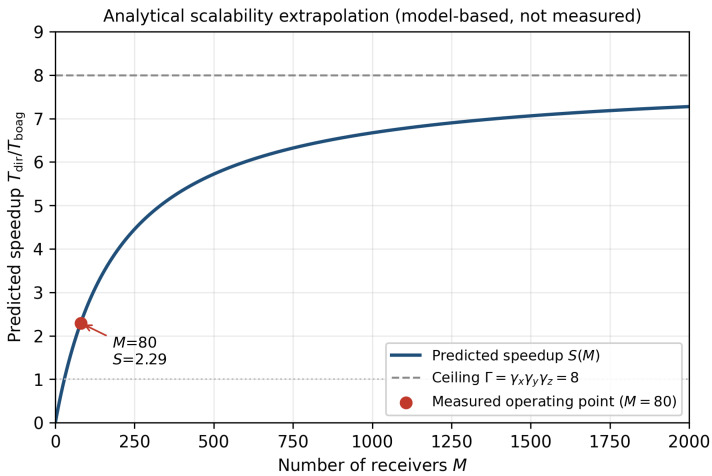
Analytical scalability extrapolation. The curve is a model-based prediction of speedup versus receiver count *M*, calibrated from the single measured operating point (red marker) under the assumption $c_{\mathrm{dir}}\approx c_{\mathrm{blk}}$; it is not additional measured data. The dashed line marks the theoretical ceiling $\Gamma =\gamma_{x}\gamma_{y}\gamma_{z}=8$, which the predicted speedup approaches but cannot exceed.

To make this concrete, we calibrate $c_{\mathrm{dir}}/c_{\mathrm{blk}}\approx 1$ (i.e., the two per-voxel costs are assumed comparable) and solve for the remaining ratio $r=c_{\mathrm{interp}}/c_{\mathrm{blk}}$ from the single measured point (M=80, Γ=8, S=2.29), giving r≈24.9. Figure 9 plots the resulting curve S(M). At the tested configuration, the method operates well below its ceiling of Γ=8; the model predicts that increasing *M* into the hundreds would move the operating point substantially closer to that ceiling, while the achievable speedup at M=80 (2.29×) is already consistent with a regime where the fixed per-voxel interpolation cost is a non-negligible fraction of the total cost. We emphasize that the specific calibrated curve depends on the assumption $c_{\mathrm{dir}}\approx c_{\mathrm{blk}}$ and on a single data point; confirming it quantitatively would require benchmarking at a small number of additional values of *M*, which we leave for future work. The two structural conclusions above—invariance to *P*, and a hard ceiling at Γ that is approached monotonically as *M* grows—do not depend on this calibration and follow directly from the complexity model in (28).

The accuracy of the proposed receiver-domain decomposition method depends critically on the interpolation of low-resolution subimages. In this section, we quantify the approximation error introduced by interpolating phase-compensated block images from a coarse spatial grid to a fine grid.

#### 6.2.1. Problem Formulation

Recall that the coherent block image associated with receiver subset $S_{k}$ is given by

(30) $$G_{k}\left(p\right)=a_{k}^{H}\left(p\right)y_{k},$$

where $a_{k}\left(p\right)$ is the steering vector restricted to the receivers in subset $S_{k}$, and $y_{k}$ is the corresponding compressed data vector.

To enable interpolation, we introduce a reference phase

(31) $$\phi_{k}^{\mathrm{ref}}\left(p\right)=2\pi f_{c}\tau_{k}^{\mathrm{ref}}\left(p\right),$$

where $\tau_{k}^{\mathrm{ref}}\left(p\right)$ is defined using the centroid of the receiver subset. The phase-compensated field is then

(32) $${\tilde{G}}_{k}\left(p\right)=G_{k}\left(p\right)e^{-j\phi_{k}^{\mathrm{ref}}\left(p\right)}.$$

The key idea is that ${\tilde{G}}_{k}\left(p\right)$ exhibits significantly reduced oscillatory behavior compared to $G_{k}\left(p\right)$, making it suitable for interpolation.

#### 6.2.2. Interpolation Approximation

Let $\Omega_{f}$ denote the fine grid and $\Omega_{c}\subset \Omega_{f}$ a coarse grid with spacings $\left(c_{x},c_{y},c_{z}\right)$. Let ${\tilde{G}}_{k}^{\left(c\right)}$ denote samples of ${\tilde{G}}_{k}$ on $\Omega_{c}$. The interpolated approximation from the coarse grid (c) to the fine grid (f) is

(33) $${\hat{G}}_{k}\left(p\right)=I_{c}^{f}\left({\tilde{G}}_{k}^{\left(c\right)}\right)\left(p\right)e^{j\phi_{k}^{\mathrm{ref}}\left(p\right)},$$

where $I_{c}^{f}$ denotes trilinear interpolation.

#### 6.2.3. Error Bound

We now quantify the error introduced by this approximation.

**Proposition** **1.** *Assume that the phase-compensated field ${\tilde{G}}_{k}\left(p\right)$ belongs to $C^{2}\left(\Omega \right)$, and that all second-order partial derivatives are uniformly bounded*

(34) $$\left|\frac{\partial^{2}{\tilde{G}}_{k}}{\partial x_{i}\partial x_{j}}\left(p\right)\right|\leq M_{k},\;\forall p\in \Omega .$$

*Then the interpolation error satisfies*

(35) $$\left|{\tilde{G}}_{k}\left(p\right)-I_{c}^{f}\left({\tilde{G}}_{k}^{\left(c\right)}\right)\left(p\right)\right|\leq C_{k}\left(c_{x}^{2}+c_{y}^{2}+c_{z}^{2}\right),$$

*for all p∈Ω, where $C_{k}$ depends on the derivative bounds.*

**Proof.**  The result follows from standard interpolation error bounds for trilinear interpolation applied independently to the real and imaginary parts of ${\tilde{G}}_{k}\left(p\right)$. Since ${\tilde{G}}_{k}$ is twice continuously differentiable, the interpolation error is proportional to the second derivatives and scales quadratically with the grid spacing. □

Since multiplication by the unit-modulus phase factor $e^{j\phi_{k}^{\mathrm{ref}}\left(p\right)}$ does not affect the magnitude of the error, the same bound applies to the reconstructed field

(36) $$|G_{k}\left(p\right)-{\hat{G}}_{k}\left(p\right)|\leq C_{k}\left(c_{x}^{2}+c_{y}^{2}+c_{z}^{2}\right).$$

Summing over all receiver blocks yields the global bound

(37) $$|G\left(p\right)-\hat{G}\left(p\right)|\leq \left(\sum_{k=1}^{K}C_{k}\right)\left(c_{x}^{2}+c_{y}^{2}+c_{z}^{2}\right).$$

#### 6.2.4. Role of Phase Compensation

The validity of the above bound depends critically on the smoothness of the compensated field ${\tilde{G}}_{k}\left(p\right)$. Without phase compensation, the block field is

(38) $$G_{k}\left(p\right)=a_{k}^{H}\left(p\right)y_{k}=\sum_{m\in S_{k}}e^{j2\pi f_{c}\tau_{m}\left(p\right)}\;y_{m},$$

which generally exhibits rapid oscillation with respect to p due to the carrier-phase term. After reference-phase compensation, the block field becomes

(39) $${\tilde{G}}_{k}\left(p\right)=G_{k}\left(p\right)e^{-j\phi_{k}^{\mathrm{ref}}\left(p\right)}=\sum_{m\in S_{k}}y_{m}\;e^{j2\pi f_{c}\left(\tau_{m}\left(p\right)-\tau_{k}^{\mathrm{ref}}\left(p\right)\right)},$$

so that the dominant oscillatory component is removed and the residual field varies more slowly with p. This reduction in oscillation directly reduces the constants $C_{k}$ in the interpolation error bound, enabling accurate interpolation on a coarse grid.

#### 6.2.5. Validity of the Smoothness Assumption

The error bound in Proposition 1 rests on the assumption that the phase-compensated field ${\tilde{G}}_{k}\left(p\right)$ belongs to $C^{2}\left(\Omega \right)$ with uniformly bounded second-order derivatives. We now discuss when this assumption holds in practice and when it is expected to break down.

When the assumption holds. From (39), the compensated block field is a coherent sum of terms of the form $y_{m}e^{j2\pi f_{c}\left(\tau_{m}\left(p\right)-\tau_{k}^{\mathrm{ref}}\left(p\right)\right)}$. Each residual phase $\tau_{m}\left(p\right)-\tau_{k}^{\mathrm{ref}}\left(p\right)$ represents the bistatic delay deviation of receiver *m* from the subaperture centroid. When the receivers in $S_{k}$ are spatially compact relative to the target range, this deviation is small and varies smoothly with p, so ${\tilde{G}}_{k}$ inherits the smoothness of the bistatic delay function. In this regime, the $C^{2}$ assumption is well justified and the bound (35) is tight.

Effect of subaperture size. As the subaperture $S_{k}$ grows larger, the residual phase deviations $\tau_{m}\left(p\right)-\tau_{k}^{\mathrm{ref}}\left(p\right)$ increase, and ${\tilde{G}}_{k}$ becomes more oscillatory. In the extreme case where all *M* receivers form a single block (K=1), no phase compensation is possible and the uncompensated field (38) oscillates at the carrier frequency scale, violating the smoothness assumption entirely. This motivates using a sufficiently large number of blocks *K*, at the cost of increased coarse-grid computation.

Effect of target range. At short range, the bistatic geometry varies rapidly across the receiver array and the residual phase deviations are larger for a given subaperture size. The smoothness assumption therefore becomes more restrictive at close range, and smaller subapertures (larger *K*) or a finer coarse grid may be required to maintain accuracy. At long range the geometry is more slowly varying and the assumption is easier to satisfy.

Breakdown regimes. The smoothness assumption can fail in at least three practical scenarios. First, if the subapertures are too large, the residual oscillation after phase compensation remains rapid and $C_{k}$ in (35) is large, making the coarse grid too coarse to capture the field. Second, for extended targets or dense multipoint scatterers, the received signal $y_{k}$ contains contributions from multiple spatial locations, each with its own phase structure; the resulting ${\tilde{G}}_{k}$ may not be well described by a single smooth function. Third, at very high carrier frequencies, the phase term $2\pi f_{c}\left(\tau_{m}-\tau_{k}^{\mathrm{ref}}\right)$ oscillates more rapidly for a given geometry, tightening the constraint on subaperture size. These failure modes are consistent with the degradation regimes identified in the numerical results.

#### 6.2.6. Practical Implications

The error bound highlights three key design considerations:

- Grid spacing: The approximation error scales quadratically with the coarse-grid spacing.
- Receiver partitioning: Smaller receiver blocks improve the accuracy of the reference phase model and reduce residual oscillation.
- Phase compensation: Proper phase correction is essential; without it, interpolation becomes unreliable due to rapid phase variation.

These observations are consistent with the numerical results, which show that phase compensation significantly smooths the low-resolution block images and improves interpolation accuracy.

### 6.3. Interpretation and Practical Recommendations

The results support several conclusions. First, in the static known-envelope case, time compression makes the direct matched filter particularly efficient, so an approximation is unlikely to be beneficial on very small 3D grids. Second, phase correction is essential; without it, the coarse block fields remain highly oscillatory and interpolation is ineffective. Third, the main value of the approximation idea in this setting lies not in small proof-of-concept problems but in larger-scale images where full-grid matched filtering becomes substantially more expensive.

The method is expected to degrade in at least three regimes. First, if the uncompensated or only weakly compensated block images remain highly oscillatory, the smoothness assumptions behind interpolation are violated. Second, overly aggressive coarse-grid spacing can remove local peak structure and distort the matched-filter surface. Third, strong extended-target interference or dense multipoint scattering may produce block fields whose behavior cannot be accurately represented by simple coarse-grid interpolation without adaptive refinement.

The present study compares the proposed method against direct matched filtering only. A direct numerical comparison against other acceleration strategies for irregular or sparse apertures—such as fast factorized back-projection [6], backprojection of moving targets [7], or phase-controlled interpolation methods more broadly [11]—as well as scalability experiments over larger imaging volumes, receiver counts, and target/noise scenarios, is left for future work.

## 7. Conclusions

A receiver-domain-decomposition principle has been developed for near-field multistatic matched filtering with a known signal envelope and stationary target. The revised formulation reduces the problem to coherent spatial matched filtering of time-compressed data, which provides a clean baseline for evaluating phase-corrected interpolation. The study confirms that phase correction smooths low-resolution block images and is therefore the essential enabler for interpolation-based acceleration. An analytical scalability extrapolation, calibrated from the reported experiment, further indicates that the achievable speedup is expected to persist as the imaging volume grows and to improve monotonically, up to a bound set by the coarse-grid decimation factor, as the number of receivers increases. At the same time, the experiments show that small static problems can favor direct matched filtering because the interpolation overhead dominates. The proposed framework is therefore best viewed as a scalable strategy for larger search spaces and more demanding configurations rather than as a universal replacement for direct computation.

## Figures and Tables

**Table 1:** Summary of principal notation.

Symbol	Definition
Arrays & Data	
$x_{m}\left[n\right]$, x[n]	Received signal; snapshot vector
$X\in C^{M\times N}$	Full data matrix
$w_{m}\left[n\right]$	Additive Gaussian noise
a(p)	Steering vector
$y=X\bar{d}$	Time-compressed data
Image Fields & Scores	
G(p), Q(p)	Matched field; direct score
$G_{k}\left(p\right)$	Block matched field
${\tilde{G}}_{k}\left(p\right)$	Phase-compensated block field
${\hat{G}}_{k}\left(p\right)$, $\hat{Q}\left(p\right)$	Interpolated field; approximate score
Receiver Decomposition	
*K*, $S_{k}$	No. blocks; *k*-th receiver subset
$a_{k}\left(p\right)$, $y_{k}$	Block steering vector; block data
Complexity & Error	
$T_{\mathrm{dir}}$, $T_{\mathrm{app}}$	Direct/approximation runtimes
$M_{k}$, $C_{k}$	Derivative bound; error constant
$\Delta_{\mathrm{rms}}$, $\Delta_{\max}$	Normalized RMS/max error
Geometry & Signal Model	
$p_{\mathrm{tx}}$	Transmitter location
$q_{m}$	*m*-th receiver location
p, $p_{★}$	Candidate point; true target
*M*, *N*	No. receivers; temporal samples
*c*, $f_{c}$	Propagation speed; carrier freq.
α	Unknown target amplitude
d[n], d	Signal envelope; conjugated vector
Delays & Phases	
$\tau_{m}\left(p\right)$	Bistatic delay, receiver *m*
${\bar{q}}_{k}$	Centroid of subset $S_{k}$
$\tau_{k}^{\mathrm{ref}}\left(p\right)$	Reference delay via centroid
$\phi_{k}^{\mathrm{ref}}\left(p\right)$	Reference phase $2\pi f_{c}\tau_{k}^{\mathrm{ref}}$
Grids & Interpolation	
$\Omega_{f}$, $\Omega_{c}$	Fine grid; coarse grid
$\left(c_{x},c_{y},c_{z}\right)$	Coarse-grid spacings
$\left(\gamma_{x},\gamma_{y},\gamma_{z}\right)$	Decimation factors
*P*, $P_{c}$	Fine/coarse voxel counts
$I_{c}^{f}$	Trilinear interpolation operator

## Data Availability

The data presented in this study are available from the corresponding author upon reasonable request.

## References

[1] Mensa D.L. (1991). High Resolution Radar Cross Section Imaging.

[2] Soumekh M. (1992). A system model and inversion for synthetic aperture radar imaging. IEEE Trans. Image Process..

[3] Nilsson S., Andersson L.E. Application of fast backprojection techniques for some inverse problems of synthetic aperture radar. Proceedings of the SPIE, Algorithms for Synthetic Aperture Radar Imagery V.

[4] Boag A. (2001). A fast multilevel domain decomposition algorithm for radar imaging. IEEE Trans. Antennas Propag..

[5] Saurer M.M., Na H., Brinkmann M., Eibert T.F. (2025). On the solution of linearized inverse scattering problems in near-field microwave imaging by operator inversion and matched filtering. IEEE Trans. Microw. Theory Tech..

[6] Ulander L.M.H., Hellsten H., Stenstrom G. (2003). Synthetic-aperture radar processing using fast factorized back-projection. IEEE Trans. Aerosp. Electron. Syst..

[7] Gaibel A., Boag A. (2021). Backprojection imaging of moving objects. IEEE Trans. Antennas Propag..

[8] Song T., Yao X., Wang L., Wang Y., Sun G. (2024). Fast factorized Kirchhoff migration algorithm for near-field radar imaging with sparse MIMO arrays. IEEE Trans. Geosci. Remote. Sens..

[9] Liu Y., Tao M., Shi T., Wang J., Wang J., Mao X. (2024). Sub-aperture polar format algorithm for curved trajectory millimeter wave radar imaging. IEEE Trans. Radar Syst..

[10] Boag A., Bresler Y., Michielssen E. (2000). A multilevel domain decomposition algorithm for fast *O*(*N*^2^log*N*) reprojection of tomographic images. IEEE Trans. Image Process..

[11] Wahl D.E., Eichel P.H., Ghiglia D.C., Jakowatz C.V. (1994). Phase gradient autofocus—A robust tool for high resolution SAR phase correction. IEEE Trans. Aerosp. Electron. Syst..

[12] Haimovich A.M., Blum R.S., Cimini L.J. (2008). MIMO Radar with Widely Separated Antennas. IEEE Signal Process. Mag..

[13] Li J., Stoica P. (2009). MIMO Radar Signal Processing.

[14] Price G.A.J., Moate C., Andre D., Yuen P. (2023). Sidelobe Suppression Techniques for Near-Field Multistatic SAR. Sensors.

[15] Masoodi M., Gennarelli G., Noviello C., Catapano I., Soldovieri F. (2025). Performance Assessment of Multistatic/Multi-Frequency 3D GPR Imaging by Linear Microwave Tomography. Sensors.

[16] Cheng Q., Zhang Y., Zeng C., Zhou Z., Liao G., Tao H. (2025). Near-Field Target Detection with Range–Angle-Coupled Matching Based on Distributed MIMO Radar. Sensors.

[17] Molaei A.M., Hu S., Skouroliakou V., Fusco V., Chen X., Yurduseven O. (2023). Fast Image Reconstruction for Near-Field Terahertz Imaging with Multistatic Non-Uniform Sparse Arrays. Proc. SPIE.

[18] Ivanenko Y., Vu V.T., Batra A., Kaiser T., Pettersson M.I. (2022). Interpolation Methods with Phase Control for Backprojection of Complex-Valued SAR Data. Sensors.

[19] Lu S., Liu F., Li Y., Zhang K., Huang H., Zou J., Li X., Dong Y., Dong F., Zhu J. (2024). Integrated Sensing and Communications: Recent Advances and Ten Open Challenges. IEEE Internet Things J..

[20] Wang J., Aubry P., Yarovoy A. (2020). 3-D short-range imaging with irregular MIMO arrays using NUFFT-based range migration algorithm. IEEE Trans. Geosci. Remote. Sens..

[21] Brinkmann M., Hamberger G.F., Eibert T.F. Nearfield multiple-input multiple-output inverse synthetic aperture radar for high-resolution imaging of large objects. Proceedings of the European Radar Conference (EuRAD).

[22] Li S., Zhao G., Li H., Ren B., Hu W., Liu Y., Yu W., Sun H. (2015). Near-field radar imaging via compressive sensing. IEEE Trans. Antennas Propag..

[23] Manisali I., Oral O., Oktem F.S. (2024). Efficient Physics-Based Learned Reconstruction Methods for Real-Time 3D Near-Field MIMO Radar Imaging. Digit. Signal Process..

[24] Oral O., Oktem F.S. (2024). Plug-and-play regularization on magnitude with deep priors for 3D near-field MIMO imaging. IEEE Trans. Comput. Imaging.

